# Lung cancer associated with partial anomalous pulmonary venous connection in the non-resected lobe: A case report

**DOI:** 10.1016/j.ijscr.2024.110454

**Published:** 2024-10-12

**Authors:** Yuuki Matsui, Koji Takami

**Affiliations:** Department of General Thoracic Surgery, NHO Osaka National Hospital, Osaka, Japan

**Keywords:** Partial anomalous pulmonary venous connection, Congenital vascular anomaly, Right heart failure, Lung cancer

## Abstract

**Introduction and importance:**

Partial anomalous pulmonary venous connection (PAPVC) is a relatively rare congenital vascular anomaly that complicates the surgical management of lung cancer and other lung lesions.

**Case presentation:**

A 74-year-old woman underwent computed tomography (CT) during an episode of chest trauma. Chest CT showed a 24-mm ground glass opacity in the right lower lobe and a PAPVC in the right upper lobe. She was diagnosed with suspected stage IA1 lung cancer with clinical T1miN0M0 and was scheduled for surgery. Preoperative catheterization revealed a pulmonary to systemic flow ratio (Qp/Qs) of 0.98. Surgical repair of PAPVC is indicated when the patient is symptomatic and has a Qp/Qs of 1.5 to 2.0 or more. The patient was scheduled for right lower lobectomy. Although worsening of postoperative right heart strain was considered, the Qp/Qs results indicated that surgical repair of the PAPVC was unnecessary. The intraoperative circulatory dynamics remained stable, and a right lower lobectomy was performed. Her postoperative course was uneventful. There was no evidence of right heart failure or recurrence of lung cancer at the last follow-up examination 6 and 12 months after surgery.

**Clinical discussion:**

If the PAPVC is in the non-resected lobe, preoperative assessment is really important, as major lung resection can increase shunt flow and cause right heart failure.

**Conclusion:**

We believe that careful interpretation of CT images of all pulmonary veins before major lung resection while considering PAPVC is important for safe perioperative management and adequate evaluation of cardiac dynamics when PAPVC is present.

## Highlights

• A partial anomalous pulmonary venous connection (PAPVC) is a congenital vascular anomaly seen in 0.4-0.7% of patients.
• When PAPVR is present in the non-resected lobe, lobectomy, the standard procedure for lung cancer, may cause postoperative right heart failure.
• If a PAPVC is present in the non-resected lobe, it is necessary to assess the risk of right heart failure and evaluate the need for revascularization.

## 1 Introduction

Partial anomalous pulmonary venous connection (PAPVC) is a relatively rare congenital anomaly, with a reported incidence of 0.4–0.7 % in the general population at autopsy [1]. If the PAPVC is in the non-resected lobe, major lung resection can increase the volume of shunt flow and cause right heart failure. Therefore, it is very important to confirm the presence of PAPVC before surgery.

We herein report a case in which we successfully detected lung cancer in the right lower lobe and a PAPVC of the right superior pulmonary vein on preoperative CT, and performed right lower lobectomy after the evaluation of cardiac dynamics with right heart catheterization.

The work has been reported in line with the SCARE criteria and the revised 2023 SCARE guidelines [2].

## 2 Case presentation

**Fig. 1:**
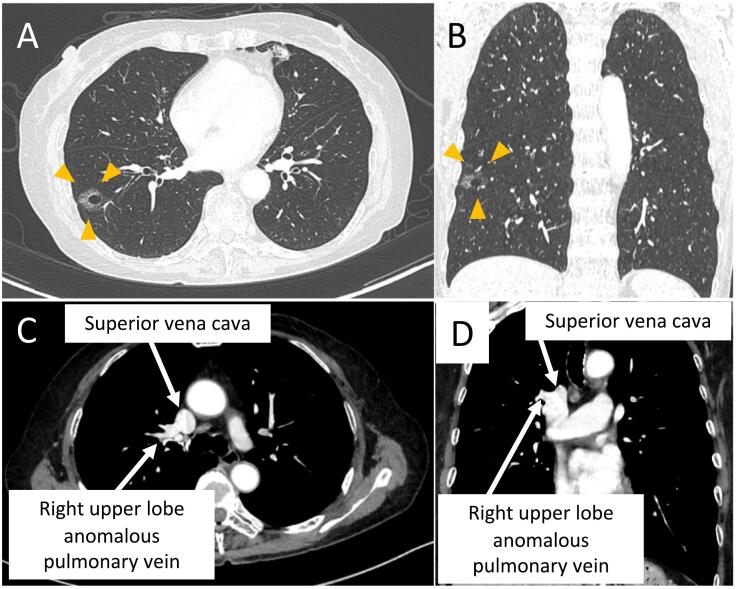
Computed tomography images. (A, B) Thoracic computed tomography showed a tumor shadow indicated by yellow arrows in the right lower lobe and (C, D) an enhanced anomalous pulmonary vein. (For interpretation of the references to colour in this figure legend, the reader is referred to the web version of this article.)

**Fig. 2:**
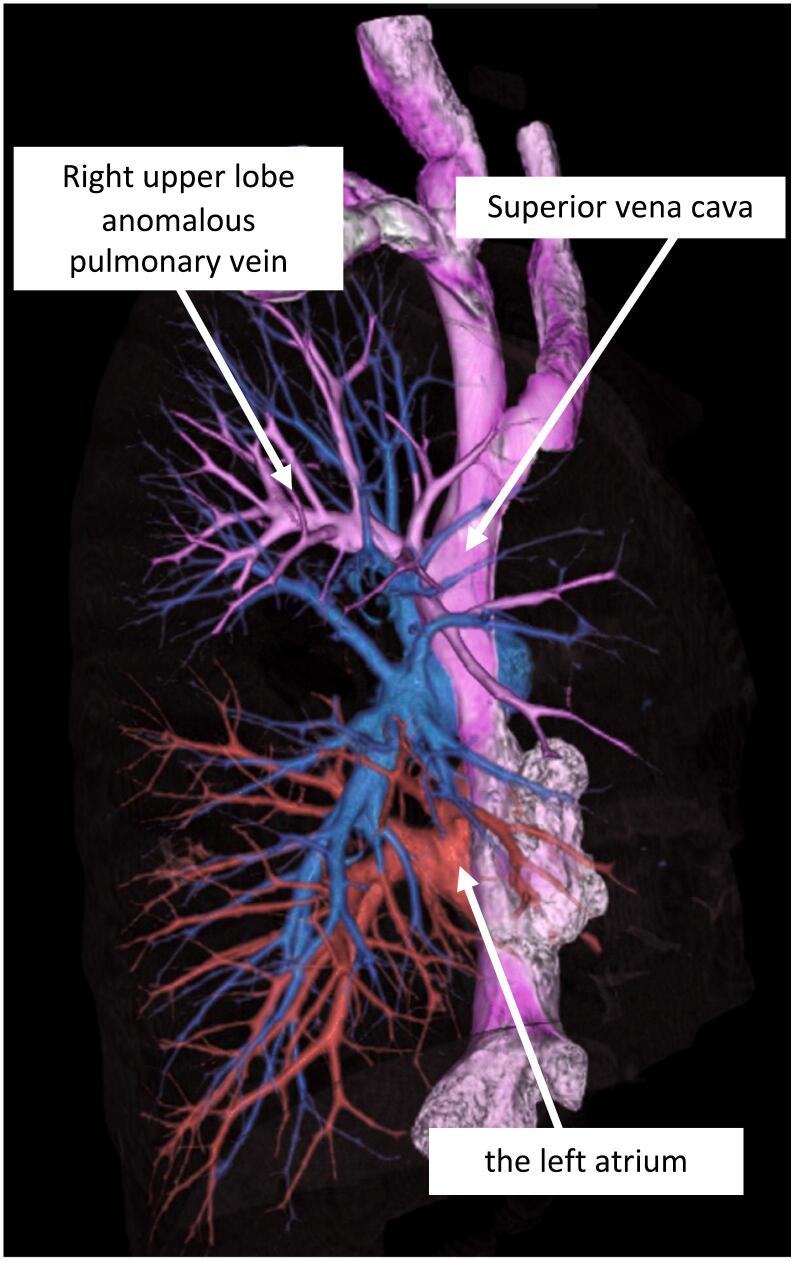
Three-dimensional computed tomography. A 3D computed tomography image of the pulmonary vessels showed the absence of a right upper lobe pulmonary vein draining into the left atrium, and a partial anomalous pulmonary venous connection consisting of drainage of the right upper lobe pulmonary vein into the superior vena cava.

**Fig. 3:**
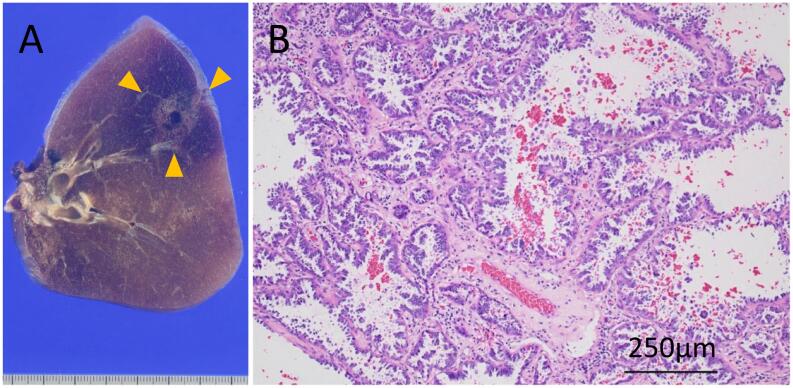
The histopathological findings. (A) Macroscopic findings of the formalin-fixed resection specimen. Macroscopic findings showed a cavitary tumor with a maximum diameter of 2.1 × 1.5 cm and invasive diameter of 1.5 cm in the right lower lobe. The tumor extent is indicated by yellow arrows. (B) Hematoxylin and Eosin stained microscopic image revealed a papillary adenocarcinoma. (For interpretation of the references to colour in this figure legend, the reader is referred to the web version of this article.)

An asymptomatic 74-year-old woman presented with a right lower lobe pulmonary shadow that was incidentally found on chest computed tomography (CT) performed to investigate chest trauma. CT revealed a 24-mm ground glass opacity with a cavity in the right lower lobe and a PAPVC in the right upper lobe (Fig. 1). The PAPVC involved drainage of the right upper lobe pulmonary vein into the superior vena cava (SVC) (Fig. 2). A preoperative transbronchial biopsy was not performed because bronchus leading to the tumor could not be identified on CT. We therefore planned intraoperative fine-needle aspiration cytology. Echocardiography did not show atrial septal defects nor abnormal cardiovascular activity. The patient was asymptomatic, and a selective pulmonary artery occlusion study in cardiac catheterization revealed a pulmonary to systemic flow ratio (Qp/Qs) of 0.98. Pulmonary function tests showed a forced expiratory volume in the first second of 1.96 L and forced vital capacity of 2.73 L. The patient was clinically diagnosed with T1miN0M0 stage IA1, and lobectomy was deemed appropriate based on the cancer profile. The risk of right heart failure due to right lower lobectomy was determined to be low from the perspective of circulatory dynamics, and a right lower lobectomy without PAPVC repair was planned. Based on intraoperative fine-needle aspiration cytology, the lesion was suspected to be a non-small cell lung cancer. The patient was curatively treated by right lower lobectomy and lymph -node dissection. A formalin-fixed resected specimen showed a cavitary tumor with a maximum diameter of 2.1 cm in the right lower lobe. Histopathological examination revealed a primary lung adenocarcinoma (Fig. 3). The final histopathological diagnosis was a papillary adenocarcinoma with a maximum diameter of 2.1 × 1.5 cm and invasive diameter of 1.5 cm, at pathological stage IA2 with T1bN0M0. Her postoperative course was uneventful. Follow-up echocardiography 6 months after surgery showed no increase in right heart load. The patient has been in good health without recurrence or cardiovascular events for 12 months postoperatively.

## 3 Discussion

Partial anomalous pulmonary venous connection (PAPVC) is a relatively rare congenital anomaly, with a reported incidence of 0.4–0.7 % in the general population at autopsy [1]. Since it has been reported that patients with PAPVC are often asymptomatic [3], there are many undiagnosed cases and the actual incidence rate may be higher. Although there are still few reports on patients with lung cancer and PAPVC, there are some reported criteria for treatment eligibility when patients with lung cancer and PAPVC undergo surgery.

It has been reported that surgical repair of PAPVC is indicated for symptomatic patients with Qp/Qs >1.5 [4], and for asymptomatic patients with Qp/Qs >2.0, regardless of associated cardiac defects [5]. In practice, it has been reported that PAPVC patients rarely show clinical symptoms [3]; however, PAPVC may cause serious problems in lung cancer patients undergoing surgery. If the PAPVC is located in the resected lobe with lung cancer, major lung resection can be the treatment option. However, if the PAPVC is present in the non-resected lobe, major lung resection increases shunt inflow and may cause right heart failure. Therefore, the assessment of cardiac dynamics by preoperative right heart catheterization is important to prevent heart failure and evaluate the need for surgical repair of PAPVC. Since our patient's Qp/Qs was <1.5 in this case, it was determined that PAPVC repair was not necessary.

Many operative procedures for the correction of PAPVC have been reported [[6], [7], [8], [9]], and treatment strategies depend on the location of the PAPVC. A left-sided PAPVC can be corrected by repair, such as apical anastomosis to a transected normal pulmonary vein or direct apical anastomosis to the left auricle or left atrium. However, in a right-sided PAPVC such as in this case, the abnormal vein returning to the superior vena cava (SVC) is short and often requires repair with an artificial heart-lung machine. There are also reports of the usefulness of a new surgical technique with minimum right atriotomy and double-barreled arrangement of systemic and pulmonary venous channels (double-decker technique) for the repair of right-sided PAPVC [10].

In a previous report, there were 17 cases of PAPVC in the non-resected lobe among lung cancer patients undergoing surgery [4,7,11,12], including the present case. Regarding the location of lung cancer and PAPVC, 13 cases were ipsilateral and 4 were contralateral. In most cases, the PAPVC is found in the pulmonary vein of the upper lobe, which often returns to the SVC or azygos vein on the right side, and to the brachiocephalic vein on the left side [13]. Therefore, PAPVC remnants can occur bilaterally. Furthermore, since postoperative deaths due to right heart failure caused by lung resection other than the one in which the contralateral PAPVC is present have also been reported [6], it is necessary to attend to the bilateral pulmonary vein. Therefore, we consider that it is important to carefully check where the right and left upper lobe pulmonary veins return when performing a lower lobectomy and where the contralateral upper lobe pulmonary vein returns when performing an upper lobectomy.

## 4 Conclusion

To facilitate safe perioperative management, the possibility of a PAPVC should always be considered and cardiac dynamics should be adequately evaluated when PAPVC is present. To this end, it is very important to carefully check CT images of all pulmonary veins before major lung resection.

## Author contribution

Yuuki Matsui wrote the original draft, reviewed, and edited the manuscript. Koji Takami supervised, wrote, reviewed, and edited the manuscript. All authors have read and approved the final manuscript.

## Consent

Written informed consent was obtained from the patient for publication of this case report and accompanying images. A copy of the written consent is available for review by the Editor-in-Chief of this journal on request.

## Ethical approval

Ethics approval is not required for case reports in our institution as they are deemed not to be research.

## Guarantor

Koji Takami

## Research registration number

None.

## Funding

The authors declare that they have no disclosure of financial interests.

## Conflict of interest statement

The authors declare that they have no competing interests.
